# Patient selection, inter-fraction plan robustness and reduction of toxicity risk with deep inspiration breath hold in intensity-modulated radiotherapy of locally advanced non-small cell lung cancer

**DOI:** 10.3389/fonc.2022.966134

**Published:** 2022-08-30

**Authors:** Kristine Fjellanger, Linda Rossi, Ben J. M. Heijmen, Helge Egil Seime Pettersen, Inger Marie Sandvik, Sebastiaan Breedveld, Turid Husevåg Sulen, Liv Bolstad Hysing

**Affiliations:** ^1^ Department of Oncology and Medical Physics, Haukeland University Hospital, Bergen, Norway; ^2^ Institute of Physics and Technology, University of Bergen, Bergen, Norway; ^3^ Department of Radiotherapy, Erasmus MC Cancer Institute, Erasmus University Medical Center, Rotterdam, Netherlands

**Keywords:** Deep inspiration breath hold (DIBH), gating, lung cancer radiotherapy, radiotherapy robustness, normal tissue complication probability (NTCP), autoplanning, iCE, radiation toxicity

## Abstract

**Background:**

State-of-the-art radiotherapy of locally advanced non-small cell lung cancer (LA-NSCLC) is performed with intensity-modulation during free breathing (FB). Previous studies have found encouraging geometric reproducibility and patient compliance of deep inspiration breath hold (DIBH) radiotherapy for LA-NSCLC patients. However, dosimetric comparisons of DIBH with FB are sparse, and DIBH is not routinely used for this patient group. The objective of this simulation study was therefore to compare DIBH and FB in a prospective cohort of LA-NSCLC patients treated with intensity-modulated radiotherapy (IMRT).

**Methods:**

For 38 LA-NSCLC patients, 4DCTs and DIBH CTs were acquired for treatment planning and during the first and third week of radiotherapy treatment. Using automated planning, one FB and one DIBH IMRT plan were generated for each patient. FB and DIBH was compared in terms of dosimetric parameters and NTCP. The treatment plans were recalculated on the repeat CTs to evaluate robustness. Correlations between ΔNTCPs and patient characteristics that could potentially predict the benefit of DIBH were explored.

**Results:**

DIBH reduced the median D_mean_ to the lungs and heart by 1.4 Gy and 1.1 Gy, respectively. This translated into reductions in NTCP for radiation pneumonitis grade ≥2 from 20.3% to 18.3%, and for 2-year mortality from 51.4% to 50.3%. The organ at risk sparing with DIBH remained significant in week 1 and week 3 of treatment, and the robustness of the target coverage was similar for FB and DIBH. While the risk of radiation pneumonitis was consistently reduced with DIBH regardless of patient characteristics, the ability to reduce the risk of 2-year mortality was evident among patients with upper and left lower lobe tumors but not right lower lobe tumors.

**Conclusion:**

Compared to FB, DIBH allowed for smaller target volumes and similar target coverage. DIBH reduced the lung and heart dose, as well as the risk of radiation pneumonitis and 2-year mortality, for 92% and 74% of LA-NSCLC patients, respectively. However, the advantages varied considerably between patients, and the ability to reduce the risk of 2-year mortality was dependent on tumor location. Evaluation of repeat CTs showed similar robustness of the dose distributions with each technique.

## 1 Introduction

For patients with locally advanced non-small cell lung cancer (LA-NSCLC), only two thirds are expected to be alive after two years when immunotherapy is added to concurrent chemoradiotherapy (1). Reducing severe side effects caused by irradiation of the heart, immune cells, lungs, and esophagus could directly or indirectly improve survival (2–8).

Radiotherapy of LA-NSCLC is usually performed during free breathing (FB), with a planning margin around the tumor to ensure dose coverage in all breathing phases. As an alternative to FB, deep inspiration breath hold (DIBH) has been investigated for several tumor sites in the thorax and abdomen (9–11). DIBH is a respiratory gating technique where patients hold their breath at a specific level of inspiration during radiotherapy delivery, potentially increasing the separation between the target volume and organs at risk (OARs), and allowing smaller margins due to the elimination of breathing motion (9).

For LA-NSCLC, two planning studies have shown potential of a dosimetric benefit of treatment delivery in DIBH compared to FB for in total 42 patients, where the majority had tumors in the upper lobes (12, 13). In a VMAT planning study, significant dose reductions were found for the lungs, heart, esophagus, trachea and bronchi with DIBH compared to FB (12). Due to large variation between patients, this study recommended comparative planning, which may be challenging in clinical routine because of limitations in staff, equipment and machine capacity (11). A treatment planning study for 3D-CRT found reductions in all investigated lung dose parameters and some heart and esophagus parameters with DIBH (13). In the published planning studies comparing DIBH and FB for LA-NSCLC, manual planning was used for plan generation, which introduces a risk of inconsistent plan quality. Many studies for various tumor sites, including LA-NSCLC, have shown significant improvements in plan quality with autoplanning compared to manual planning (14–16).

Previous studies have reported encouraging patient compliance of DIBH in radiotherapy for LA-NSCLC, with small intra- and inter-breath-hold uncertainties in tumor position registered in fluoroscopic movies of liquid markers during the treatment course (17) and evaluations of consecutive CT scans at treatment planning (18). However, DIBH and FB treatments have not yet been compared regarding robustness of the dose distributions against inter-fraction anatomical variations or slow inter-fraction time trends, e.g. caused by radiation-induced anatomical changes. It is not clear whether inter-fraction variations in DIBH inspiration level and FB breathing pattern affect the dose distributions differently, and how margin reduction with DIBH affects the target dose robustness.

The aims of the current study were to dosimetrically compare FB with DIBH for LA-NSCLC patients, including inter-fraction robustness, and to investigate which patients are more likely to benefit from DIBH. For this purpose, we initiated a prospective image collection study for LA-NSCLC patients treated with intensity-modulated radiotherapy (IMRT). To avoid bias, all treatment plans were generated with automated multi-criterial treatment planning with integrated beam-angle optimization (BAO) (14), and the same autoplanning configuration was used for both FB and DIBH (19). Comparisons were made in terms of dose-volume parameters and normal tissue complication probabilities (NTCPs), both on a population basis and with focus on the effect for individual patients. For these analyses, CT scans acquired in the treatment-preparation phase were used. For assessment of dosimetric robustness against inter-fraction anatomical variations, repeat DIBH and FB CT scans (rCTs) were acquired during the fractionated treatment and used to recalculate dose. Finally, we investigated to what extent specific patient or tumor characteristics could be used to predict the best choice between treatment with DIBH or with FB for new patients, thereby avoiding unnecessary patient-specific comparative planning.

## 2 Materials and methods

### 2.1 Patients and clinical treatment

Between October 2019 and May 2022, 38 consecutive patients receiving radiotherapy with curative intent according to the protocol for LA-NSCLC at Haukeland University Hospital participated in prospective image collection for this simulation study. The study was approved by the regional committee for medical and health research ethics in Western Norway (protocol code 2019/749) and all participants gave informed consent. Clinical parameters describing the disease and condition of the patients as well as the prescribed treatments were collected.

Clinical treatments were delivered with IMRT in FB as a standard. For nine patients the oncologist chose treatment in DIBH instead, mainly due to high lung doses with FB. Thirty-three patients were treated with 6 IMRT beams. Based on patient-specific assessments, three patients had one field removed to reduce lung dose and were thus treated with 5 IMRT beams and two patients with large fields were treated with VMAT. The planning strategy and objectives are described in section S1. In accordance with national guidelines, the prescribed dose was 60 or 66 Gy for concomitant treatment and 66 or 70 Gy for sequential treatment (depending on lung function, lung dose and proximity of the brachial plexus to the PTV), all in 2 Gy fractions. The plans were normalized to the median PTV dose (PTV D_median_ = 100%). Daily CBCTs followed by table corrections with six degrees of freedom were used for on-line positioning.

To ensure high quality and consistency and avoid planner bias, the manually created clinical treatment plans were not used in this study. Instead, automated plans were generated as described in section 2.3.

### 2.2 Acquired CT scans and delineation

For each patient, a 10-phase 4DCT and three DIBH CTs were acquired for planning, and a repeated 4DCT and DIBH CT were acquired during the first week (W1) and third week (W3) of treatment. Imaging was performed on a Big Bore CT scanner (Philips Healthcare, Best, The Netherlands), using a Posirest-2 support device (Civco Radiotherapy, Coralville, USA) for fixation in the supine position with arms resting above the head. The breathing curve for the 4DCT was acquired using the Philips Bellows device. DIBH was performed with the Respiratory Gating for Scanners (RGSC) system (Varian Medical Systems, Palo Alto, USA), using a marker box placed on the sternum, 2-3 mm gating window and visual feedback. The patients practiced breath holds before image acquisition at the planning CT session.

Gross tumor volumes (GTVs) for the primary tumor and lymph nodes were delineated according to ESTRO guidelines (20). For FB planning CTs and rCTs, the OARs and GTVs were delineated on the average intensity projection (AIP) of the 4DCT, and the internal GTV (IGTV) incorporated the GTV positions in all 4DCT phases. For DIBH planning, the OARs and GTVs were delineated on one of the DIBH CTs, and the IGTV incorporated the GTV positions in the two other DIBH CTs. In W1 and W3 only one DIBH rCT was acquired, hence no IGTV was delineated. For both FB and DIBH, the clinical target volume (CTV) was defined by expanding the IGTV (or GTV) by 5 mm without extending into uninvolved organs such as bone, heart, esophagus and major vessels. A 5 mm isotropic margin from the CTV was used to define the planning target volume (PTV). As OARs, the lungs, heart, esophagus, spinal canal and brachial plexus (if relevant) were delineated according to RTOG guidelines (21).

### 2.3 Automated treatment planning

The novel in-house “iCE” system for automated multi-criterial planning with integrated BAO was used to generate all the treatment plans in this study (14). In iCE, an initial Pareto-optimal, fluence-map-optimized treatment plan is automatically created in Erasmus-iCycle, based on a wish-list tuned to reflect the clinical priorities for this patient group (22). The dose distribution is then automatically reconstructed in Eclipse (Varian Medical Systems), resulting in a deliverable plan created without manual intervention. A detailed description of iCE and the applied wish-list can be found elsewhere (14).

In this study, iCE was used to automatically generate two deliverable 6-beam IMRT plans for each patient, one on the FB and one on the DIBH planning CT, each with optimized beam angles. The applied wish-list was the same for FB and DIBH planning, reflecting the common clinical protocols. The same prescription dose as in the clinical plan was used and the plans were normalized to the median dose in the PTV, as in clinical practice. The applied Eclipse version was 16.1, the Photon Optimizer algorithm was used for optimization and the Acuros External Beam algorithm was used for dose calculation.

### 2.4 Comparison of FB and DIBH

#### 2.4.1 Dosimetric comparison based on planning CT scans

The FB and DIBH plans were compared using relevant dose-volume parameters for the PTV and OARs, the effective dose to immune cells (EDIC) given as equivalent uniform dose (see section S2.4. for details) and NTCPs (NB reference to subsection (needs numbering)). For lungs and heart, where the volume is expected to be different in FB and DIBH, we further estimated the integral dose (ID [Gy·L] = D_mean_ [Gy] · volume [L]). The mean dose to the lungs, heart and esophagus are clinically important and commonly reported parameters related to toxicity, and were therefore used for evaluation of per-patient differences.

#### 2.4.2 Robustness of the dose distribution assessed with repeat CT scans

The W1 and W3 rCTs were rigidly matched to the corresponding planning CTs, using six degrees of freedom and a volume of interest covering the PTV and surrounding skeletal structures. The FB and DIBH plans were then recalculated on the respective rCTs. For the target volume, the robustness was considered sufficient if the CTV V_95%_ was > 99%. For the OARs, the changes in dose-volume parameters from planning to each rCT were evaluated.

#### 2.4.3 NTCPs

To evaluate the clinical impact of dosimetric differences between FB and DIBH, NTCPs for RP grade ≥2, acute esophageal toxicity (AET) grade ≥2 and 2-year mortality based on heart dose (heart model) were calculated according to validated models used in the proton therapy selection framework in the Netherlands (23–26). An alternative model for 2-year mortality based on the EDIC was also applied (EDIC model) (6). A detailed description of the models is given in section S2.

### 2.5 Patient characteristics and benefit of DIBH

Correlations between the ΔNTCPs [NTCP (DIBH) – NTCP (FB)] and patient characteristics that could potentially predict the benefit of DIBH were explored, with focus on characteristics that are known before or during the planning CT session:

• Primary tumor in the upper or lower lobes
• Primary tumor in the left or right lung
• Expansion of the lungs with DIBH (relative increase in lung volume compared to FB)
• Cranio-caudal motion extension of the primary tumor in FB (breathing motion)

The tumor breathing motion was determined by deformable mapping of the primary tumor GTV from the AIP to each phase of the 4DCT, followed by visual inspection of the structures to ensure accuracy, and measuring the motion extension of the GTV center of mass.

One patient had a primary tumor extending into both the right upper and middle lobes, and was grouped with the upper lobes for this analysis.

### 2.6 Statistical analysis

Statistical analyses were performed using SPSS Statistics v. 26 (IBM Corp., Armonk, USA). The two-tailed Wilcoxon signed-rank test was used for related samples. Linear regression was used to test correlations between two continuous variables. *p*-values ≤0.05 were considered statistically significant. Percentiles were established using a weighted average method.

## 3 Results

### 3.1 Patients and anatomy

Among the 38 included patients, most had stage IIIA-IIIB disease. 29 had both a primary tumor and lymph nodes in the target volume, 1 had only lymph nodes and 8 had only a primary tumor. The primary tumor was located in the upper lobes for 20 patients and the lower lobes for 17. A summary of patient and treatment characteristics is given in **Table 1**.

**Table 1:** Patient and treatment characteristics.

Characteristic		Number of Patients
Stage	IB ^1^	1
	IIB	2
	IIIA	15
	IIIB	15
	IIIC	3
	IVA ^2^	2
Target volume	Primary tumor and lymph nodes	29
	Primary tumor only	8
	Lymph nodes only	1
Primary tumor location (lobe)	Right upper	13
	Right upper + middle	1
	Right lower	7
	Left upper	6
	Left lower	10
Smoking habits	Active smoker	15
	Previous smoker	22
	Non-smoker	1
Pulmonary comorbidity	COPD	21
	Other	1
	None	16
Prescribed dose	60 Gy	14
	66 Gy	23
	70 Gy	1
Chemotherapy	Concurrent	36
	Sequential	2
**Characteristic**	**Average**	**Range**
Age (years)	66	53-82
GTV volume (cm^3^)	115	13-1021
Overall treatment time (days)	44	39-49
Tumor motion (mm) ^3^	4	0-21

^1^This patient had an inoperable tumor due to the position in the main bronchus, and received radiotherapy according to the protocol for LA-NSCLC. ^2^These patients had a solitary brain metastasis that was treated separately, and received radiotherapy with curative intent according to the protocol for LA-NSCLC. ^3^Cranio-caudal motion of the primary tumor GTV in FB. The number of patients in each category is given for categorical variables. The average value and range is given for continuous variables.

**Figure 1:**
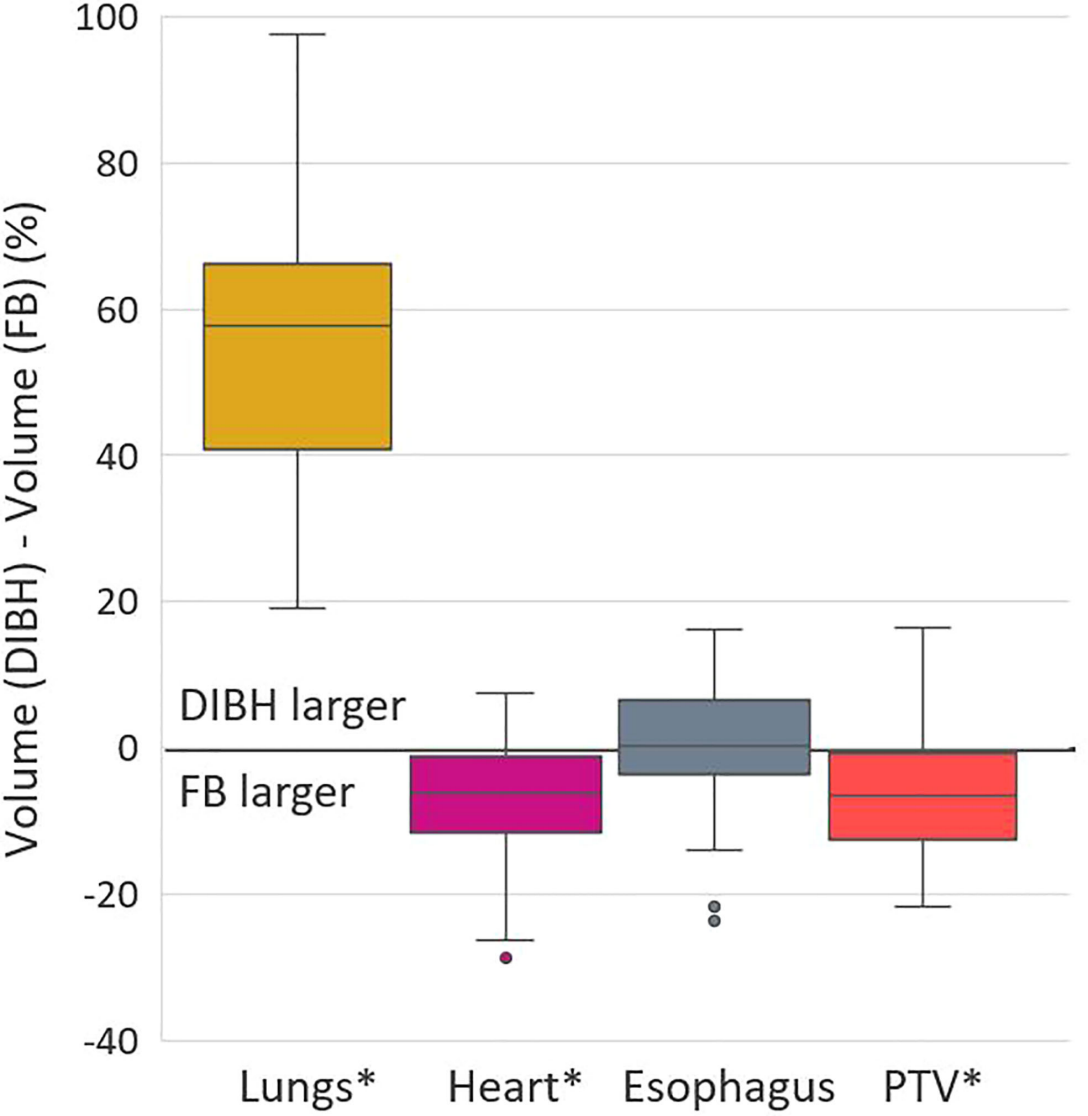
Difference in volume of structures between the FB and DIBH CTs of each patient, relative to the FB volume. Significant differences between FB and DIBH are marked with *. Boxplots show the median value (line), 1^st^ to 3^rd^ quartile (box), maximum and minimum values excluding outliers (whiskers) and outliers (dots).

At planning, the DIBH PTVs were on average 6% smaller than the FB PTVs (386 cm3 vs. 409 cm3, *p* < 0.001), the lung volumes increased by 50% (5656 cm3 vs. 3776 cm3, *p* < 0.001), and the heart volumes decreased by 7% (659 cm3 vs. 709 cm3, *p* < 0.001) with DIBH compared to FB (**Figure 1**).

### 3.2 Dosimetric comparison of FB and DIBH at planning

**Figure 2:**
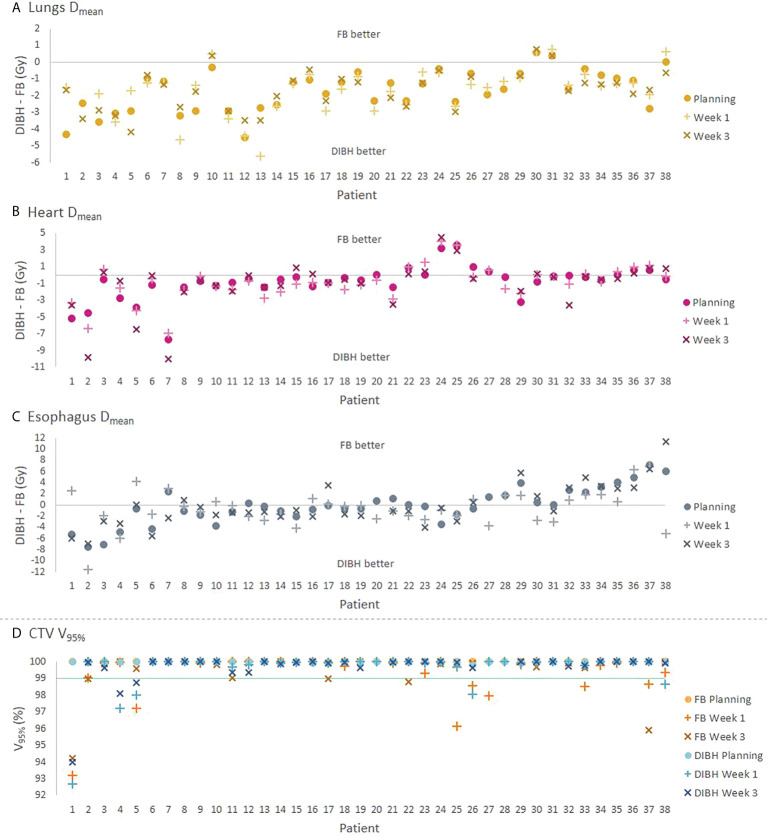
**(A–C)** Absolute differences in D_mean_ for the lungs, heart and esophagus between the DIBH and FB plans for each patient. **(D)** CTV V_95%_ for each technique, with the green line indicating the required value of 99% for the rCTs. The values at planning, W1 and W3 are represented by different symbols. For three patients where a W3 rCT was not available (patients 20, 27 and 28), results are shown only for the other time points. The patients are sorted according to the sum of DIBH-FB differences for the OARs at planning.

**Figure 3:**
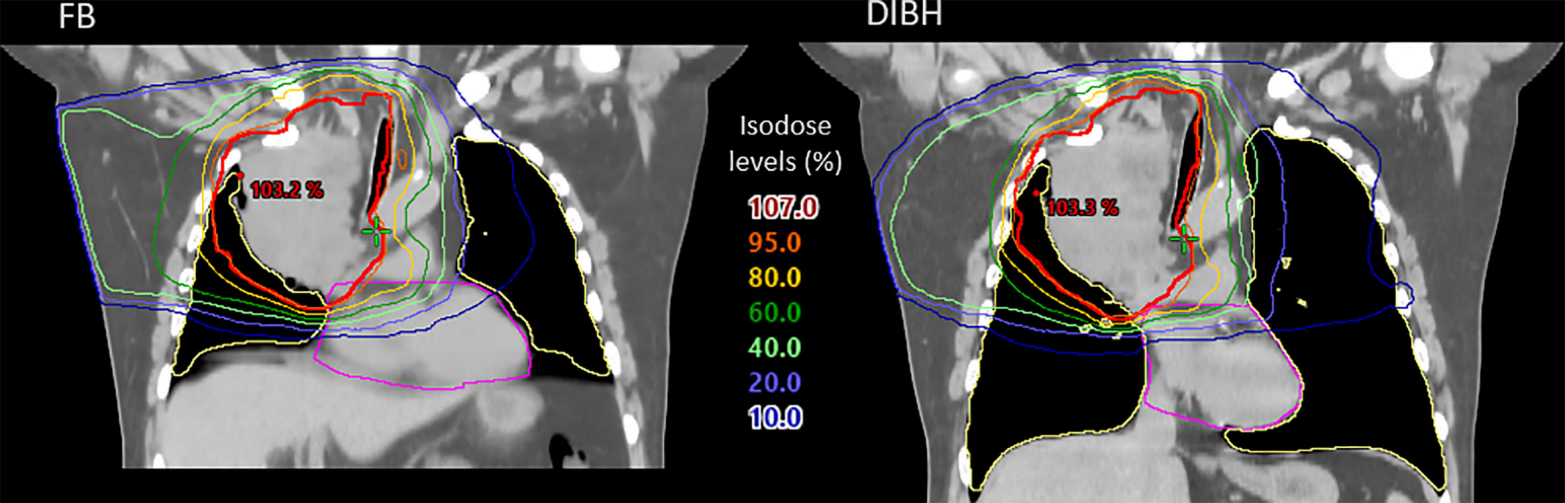
Dose distributions superimposed on planning CT scans of patient 1, showing enhanced sparing of OARs in DIBH (right) compared to FB (left). Contours are shown for the PTV (red), lungs (yellow) and heart (magenta). Isodoses are shown in percentage of the prescribed dose (60 Gy).

At planning, DIBH had a slightly lower median PTV V_95%_ than FB (**Table 2**). The objective of V_95%_ > 98% was achieved in all plans except the DIBH plans for two patients, which had V_95%_ > 95%. All dosimetric parameters for the lungs, heart and spinal canal were significantly reduced with DIBH compared to FB, except for ID to the lungs (**Table 2** and **Figure 2**). For the esophagus, no significant differences were found. There were large inter-patient variations in dosimetric differences between FB and DIBH (**Figure 2**). DIBH resulted in a lower lung D_mean_ than FB for 35/38 patients (range -4.5 to 0.6 Gy) and a lower heart D_mean_ for 28/38 patients (range -7.6 to 3.6 Gy), while for the esophagus around half the patients were better off with either technique and the difference in D_mean_ ranged from -7.5 Gy to 7.1 Gy. **Figure 3** illustrates how parts of the lungs and heart could be moved out of the treatment field with DIBH, resulting in substantial dose sparing.

**Table 2:** Dose-volume metrics and NTCPs for FB and DIBH plans at planning.

Metric	FB		DIBH		*p*-value	Patients with benefit of DIBH
	Median	10^th^–90^th^ pctl	Median	10^th^–90^th^ pctl		
PTV V_95%_ (%)	99.4	98.7–99.7	99.1	98.0–99.5	<0.001*	24%
Patient D_max_ (%)	104.9	103.9–105.9	104.7	104.0–106.1	0.8	50%
Lungs D_mean_ (Gy)	15.2	9.3–18.9	13.8	7.7–17.2	<0.001*	92%
Lungs V_5Gy_ (%)	58.7	41.4–78.7	54.3	38.0–73.5	0.007*	68%
Lungs V_20Gy_ (%)	24.9	15.5–34.1	23.7	12.8–30.9	<0.001*	89%
Lungs ID (Gy*L)	51.9	32.1-84.7	70.8	47.5-100.8	<0.001*	0%
Heart D_mean_ (Gy)	9.3	2.7–19.9	8.2	1.6–18.9	0.002 *	74%
Heart V_5Gy_ (%)	42.6	9.9–84.6	35.5	5.3–93.6	0.05 *	66%
Heart V_30Gy_ (%)	8.4	1.6–22.7	7.8	0.0–16.6	0.005*	68%
Heart ID (Gy*L)	5.6	0.4-12.7	5.2	0.3-11.5	<0.001*	71%
Esophagus D_mean_ (Gy)	19.5	10.8–30.8	19.2	13.4–30.5	0.7	55%
Esophagus V_20Gy_ (%)	36.7	23.6–55.2	36.3	26.1–56.4	0.1	39%
Esophagus V_60 Gy_ (%)	4.9	0.0–27.9	5.8	0.0–24.3	0.8	50%
Spinal canal D_max_ (Gy)	46.1	34.7–50.7	42.7	28.5–50.0	0.007 *	71%
EDIC (Gy)	4.6	2.9–6.8	4.2	2.7–6.1	<0.001*	89%
NTCP RP (%) ^1^	20.3	9.1–39.7	18.3	7.1–35.5	<0.001*	92%
NTCP AET (%) ^2^	38.9	21.9–55.4	38.8	25.3–55.9	0.8	55%
NTCP Mortality (heart) (%) ^3^	51.4	37.1–65.2	50.3	36.5–64.4	0.002*	74%
NTCP Mortality (EDIC) (%) ^4^	41.0	28.0–53.1	37.4	27.3–51.0	<0.001*	89%

^1^ Radiation pneumonitis grade ≥2, ^2^ acute esophageal toxicity grade ≥2, ^3^ 2-year mortality (heart model), ^4^ 2-year mortality (EDIC model). Median value and 10^th^–90^th^ percentile (pctl) is given, along with *p*-values for comparison between the techniques. Significant differences are marked with *. The percentage of patients with a benefit of DIBH is also given for each parameter.

### 3.3 Robustness of dose distributions assessed with rCTs

Repeat CTs in W1 were available for all 38 patients, while three patients did not complete the W3 scans due to poor condition or covid-19. The 35 patients who completed all CT sessions were included in the statistical analyses of robustness.

The target coverage in W1 and W3 was satisfactory for most patients, the median CTV V_95%_ was 100% for both FB and DIBH at all time points (Tables S3-S4), and there were no significant differences in CTV V_95%_ between FB and DIBH in neither W1 (*p* = 0.2) or W3 (*p* = 1.0). However, the CTV V_95%_ was <99% in FB for seven patients in W1 and five patients in W3, and in DIBH for five patients in W1 and three patients in W3 (**Figure 2D**).

Both for FB and DIBH, most OAR parameters were similar in the planning CT and rCTs, except for the esophagus which received a higher dose in W3 than at planning (**Tables S3, S4**, **Figure 2**). The lungs D_mean_ was also slightly increased in W3 with both techniques. The dose to the lungs and heart remained significantly lower with DIBH than FB at all time points. Per-patient analysis showed that for the lungs and heart, the D_mean_ and difference in D_mean_ between FB and DIBH were quite stable for the different time points for most patients, while for the esophagus they varied more between planning and rCTs with changes in D_mean_ of up to 8-10 Gy seen in some of the FB plans (**Figures S2** and **2**).

### 3.4 NTCPs

In addition to dose-volume parameters, a number of clinical parameters were collected and used in the NTCP calculations (**Table 1**).

**Figure 4:**
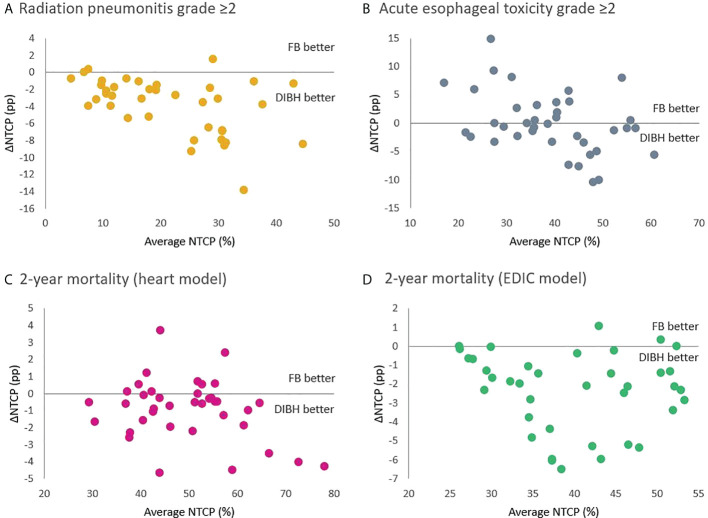
ΔNTCP between DIBH and FB as a function of the average NTCP value in the FB and DIBH plans for each patient, for **(A)** RP, **(B)** AET, **(C)** 2-year mortality (heart model) and **(D)** 2-year mortality (EDIC model). pp, percentage points.

The NTCPs for RP, 2-year mortality (heart model) and 2-year mortality (EDIC model) were significantly lower with DIBH compared to FB, with average ΔNTCPs of -3.8 percentage points (pp), -0.9 pp and -2.3 pp, respectively. There was no significant difference for AET (**Table 2**). The advantage of DIBH was generally larger for patients with higher risk of radiation-induced complication for RP (*p* = 0.002), AET (*p* = 0.01) and 2-year mortality (heart model) (*p* = 0.07) but not for 2-year mortality (EDIC model) (*p* = 0.463) (**Figure 4**).

While the 2-year mortality was lower with DIBH than FB according to both the applied models, the median NTCPs were 10-13 pp lower with the EDIC model than the heart dose model.

### 3.5 Patient characteristics and benefit of DIBH

The NTCPs for RP and 2-year mortality (EDIC model) were significantly lower with DIBH than FB regardless of tumor position, and there was no correlation between ΔNTCP and tumor motion in FB or lung expansion with DIBH (Tables S5-S6).

**Figure 5:**
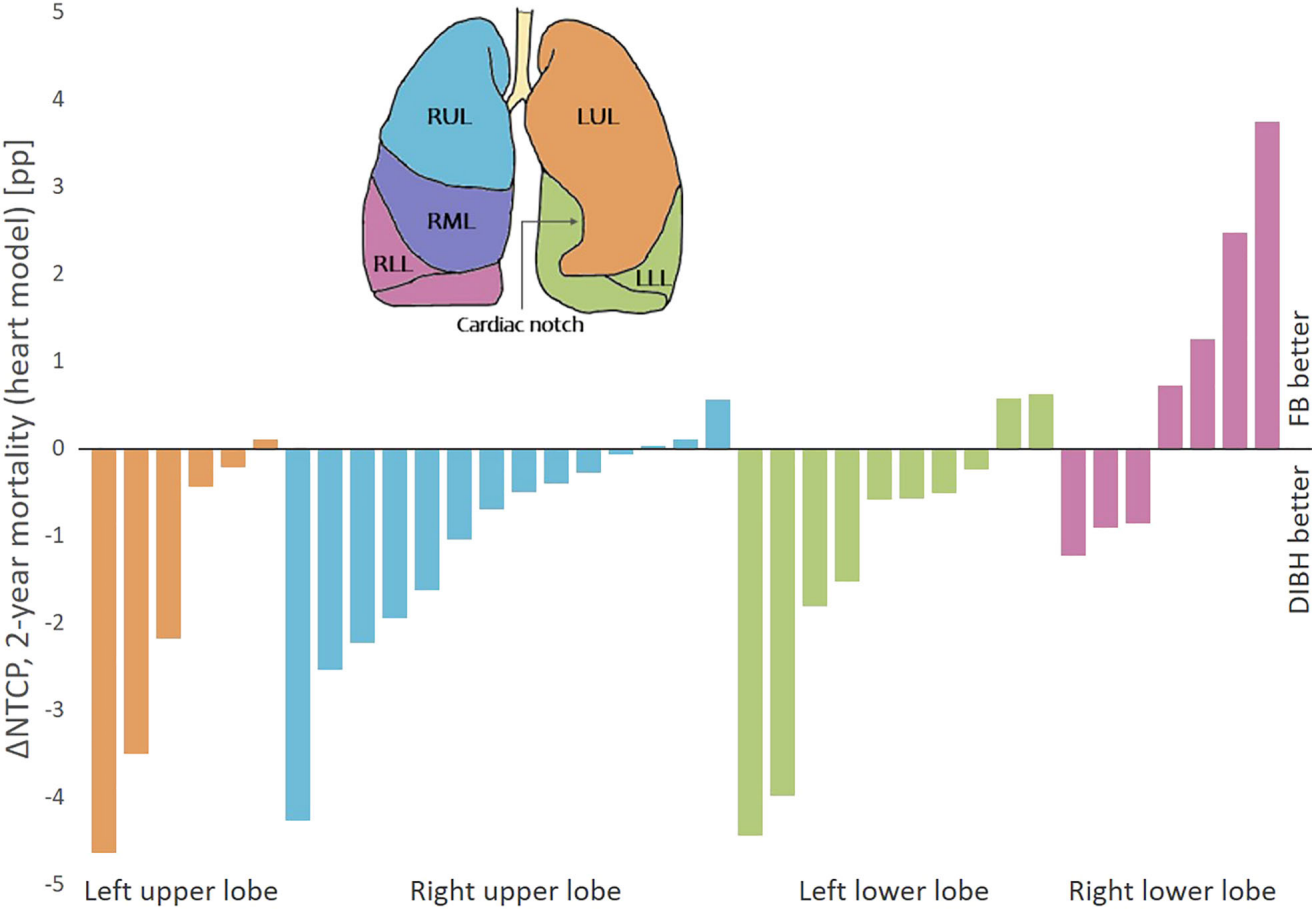
ΔNTCP for 2-year mortality (heart model) between DIBH and FB per patient, sorted according to primary tumor position. There were no patients with primary tumor in the right middle lobe. Negative ΔNTCP values are in favor of DIBH and positive values are in favor of FB. pp, percentage points.

For 2-year mortality (heart model), the NTCP was significantly lower with DIBH than FB for the patients with tumors in the upper lobe and left lung, but similar for patients with tumors in the lower lobe or right lung (Table S5). Separating the patients according to lobe, 83%, 79% and 80% of the patients with tumors in the left upper lobe, right upper lobe and left lower lobe had a lower NTCP for 2-year mortality (heart model) with DIBH than FB (ΔNTCP range -4.6 to 0.6 pp), while this benefit was seen for only 43% of the patients with tumors in the right lower lobe (ΔNTCP range -1.2 to 3.7 pp) (**Figure 5**).

## 4 Discussion

For LA-NSCLC patients treated with static beam IMRT, this study found significantly enhanced dosimetric sparing of the lungs and heart by using DIBH instead of FB. The dosimetric findings translated into reduced risks of RP and 2-year mortality. Patients with the highest complication risks benefited most from DIBH. The OAR sparing with DIBH remained similar in W1 and W3 of treatment. The robustness of the target coverage was similar for FB and DIBH, despite smaller margins in the DIBH plans. However, in 9%-14% of the DIBH plans and 14%-20% of the FB plans (depending on time point), there was not sufficient coverage of the CTV in the rCTs, suggesting a potential added value of adaptive protocols for this patient group (27).

Looking at individual patients, the sparing of the lungs with DIBH was consistent; more than 90% of the patients had a lower lung D_mean_ with DIBH than with FB. For the heart, DIBH was favorable for around 70% of the patients: for these patients the deep inspiration could likely increase the separation of the heart from the PTV and enable a dose reduction. However, for two patients, the heart D_mean_ increased by more than 3 Gy and the NTCP for 2-year mortality (heart model) by 2-4 pp with DIBH. The median values for esophagus dose were similar between FB and DIBH, but there were large inter-patient differences both between the techniques and the different time points. Changes of up to 10 Gy in the esophagus D_mean_ occurred between planning and rCTs, and the largest changes were seen in FB.

A clinical study of various respiratory gating techniques in 3D-CRT for lung cancer patients with different stages and prescriptions found less pulmonary and esophageal toxicity with respiratory gating compared to FB (28). Two previous planning studies with 3D-CRT and VMAT, although limited in the number of patients and performed with manual planning, have found an overall benefit of DIBH for LA-NSCLC patients in terms of reduced OAR doses compared to FB (12, 13). The current study showed the potential of DIBH also in static beam IMRT, in a larger and more heterogeneous cohort of LA-NSCLC patients, and also reported on inter-patient differences and robustness in terms of inter-fractional changes in delivered dose. Although both VMAT and IMRT with a few static beams are used for treating LA-NSCLC patients with intensity modulation, the latter is particularly suited for avoiding large volumes of healthy lung tissue receiving low dose (15, 29). We have also experienced such an advantage with IMRT compared to VMAT in our clinic and have therefore concluded that in our situation treatment with static beam IMRT is preferred for these patients.

Despite the promising results for OAR sparing and patient compliance, the use of DIBH for LA-NSCLC patients is still limited. In the POP-ART RT survey, the most important barriers stated for implementation or expanded use of respiratory motion management were resources in terms of equipment, staff and machine capacity (11). Identifying and prioritizing the patients with most benefit of DIBH could therefore be valuable. This study showed a consistent reduction in the NTCP for RP with DIBH compared to FB, regardless of patient characteristics. Interestingly, despite lower-lobe tumors being more mobile and therefore having more potential for margin reduction with DIBH, increased ΔNTCP for RP was not seen for neither lower-lobe compared to upper-lobe tumors, or with increased breathing motion. However, the NTCP for 2-year mortality (heart model) was reduced with DIBH for almost all patients with tumors in the upper lobes or left lower lobe, while it varied which technique was best for patients with tumors in the right lower lobe. As the mediastinum narrows during DIBH, the heart is compressed and caudal parts are moved from the left side towards the center of the body. This can increase the separation between tumors in the left lower lobe and the heart, while for tumors in the right lower lobe, decreased separation and compression of the heart could instead increase the heart dose with DIBH compared to FB. This analysis should, however, be seen as a preliminary investigation, as the number of patients in each group was small, and the interconnection between the parameters was, for this reason, not investigated. Persson et al. did not find a pattern in benefit regarding OAR doses between FB and DIBH depending on tumor position; however, their study included only three patients with lower lobe tumors, all in the left lung (12).

The NTCP models for RP, AET and 2-year mortality based on heart dose used in this study have been externally validated and carefully selected in the Dutch proton therapy selection framework (23). Recently, the radiation of immune cells and its impact on survival has received increased attention, and Jin et al. published a method for approximating the EDIC, as well as a corresponding model for 2-year overall survival based on data from the RTOG0617 study (6). When applying their model for the patients in this study, the benefit of DIBH was retained, but the estimates for median 2-year mortality were 10-13 pp lower than with the heart dose model. The EDIC model is based on one patient cohort where the dose characteristics were quite different from the ones in this study; especially the heart dose was clearly higher. In a study by Thor et al. where IMRT was applied with similar heart doses to our cohort, the estimated dose of radiation to immune cells was not found to correlate with progression-free survival (30). In our cohort, the NTCP for 2-year mortality based on EDIC seems to be driven mainly by the mean lung dose. Because this parameter was lower with DIBH as a consequence of an increase in lung volume, we cannot know the clinical relevance of a lower EDIC with DIBH and this needs further investigation.

In this study, automated treatment planning with integrated BAO was applied to achieve several benefits. Plan comparison can be performed without planner bias, and due to short planning times and very limited planner interaction, a large number of patients can be included, increasing the quality and reliability of the study. In clinical routine, autoplanning could facilitate individualized selection between FB and DIBH with virtually zero workload.

The conclusions made in this study with regard to lung sparing depend on the assumption that the same mean dose will give the same side effects for FB and DIBH, or that the expansion of the lungs distributes the functional tissue evenly. Additionally, the applied NTCP models are developed using data from FB treatment. More studies are therefore needed to determine the actual clinical benefit of DIBH in radiotherapy of LA-NSCLC.

This study has been evaluated using the RATING criteria for treatment planning studies and a score of 95% was achieved (31).

## 5 Conclusion

Compared to FB, DIBH allowed for smaller target volumes and similar target coverage. Furthermore, DIBH reduced the lung and heart dose, as well as the risks of radiation pneumonitis and 2-year mortality, for 92% and 74% of LA-NSCLC patients, respectively. The advantages of DIBH varied considerably between patients. Evaluation of rCTs showed similar robustness of the dose distributions with each technique. While DIBH reduced the risk of RP consistently regardless of patient characteristics, the ability to reduce the risk of 2-year mortality was evident among patients with upper and left lower lobe tumors but not right lower lobe tumors. Automated planning could facilitate individualized selection between FB and DIBH with no planner bias and virtually zero workload.

## Data availability statement

The data presented in this study are available on request from the corresponding author. The data are not publicly available due to privacy reasons as they are part of an ongoing study.

## Ethics statement

The study involved human participants and were reviewed and approved by the regional committee for medical and health research ethics in Western Norway (protocol code 2019/749). The patients provided their written informed consent to participate in this study.

## Author contributions

Conceptualization, KF, LR, BH, HP, SB and LH. methodology, KF, LR, BH, HP and LH. software, KF, LR, HP and SB. validation, KF. formal analysis, KF and LH. investigation, KF, IS and LH. resources, LH, BH, IS and TS. data curation, KF. writing—original draft preparation, KF. writing—review and editing, KF, LR, BH, HP, IS, TS, SB and LH. visualization, KF. supervision, LR, BH, HP and LH. project administration, LH. funding acquisition, KF and LH. All authors contributed to the article and approved the submitted version.

## Funding

This research received funding from Helse Vest RHF (grant number F-12505) and the Trond Mohn Foundation (grant number BFS2017TMT07).

## Acknowledgments

The authors thank John-Vidar Hjørnevik, Ove Dalseid and other clinical personnel at Haukeland University Hospital for their contribution to data collection.

## Conflict of interest

LR, BH and SB: Erasmus MC Cancer Institute has research collaborations with Elekta AB (Stockholm, Sweden), Accuray, Inc. (Sunnyvale, USA) and Varian Medical Systems, Inc. (Palo Alto, USA). The funders had no role in the design of the study; in the collection, analyses, or interpretation of data; in the writing of the manuscript, or in the decision to publish the results.

The remaining authors declare that the research was conducted in the absence of any commercial or financial relationships that could be construed as a potential conflict of interest.

## Publisher’s note

All claims expressed in this article are solely those of the authors and do not necessarily represent those of their affiliated organizations, or those of the publisher, the editors and the reviewers. Any product that may be evaluated in this article, or claim that may be made by its manufacturer, is not guaranteed or endorsed by the publisher.
