# Supplementary material for: Patient selection, inter-fraction plan robustness and reduction of toxicity risk with deep inspiration breath hold in intensity-modulated radiotherapy of locally advanced non-small cell lung cancer
Source: Front Oncol. 2022 Aug 30;12:966134. doi: 10.3389/fonc.2022.966134 (PMC9469652; doi:10.3389/fonc.2022.966134)
Supplement: Supplementary file 1 [file Presentation_1.pdf]

Supplementary Material

S1 Planning objectives

In the clinical treatment planning, there were maximum dose objectives for the spinal canal, brachial plexus and patient body. Additionally, the goal was to achieve sufficient target coverage and as low dose as possible to normal tissue, with the following order of priority: (1) PTV, (2) lungs, (3) heart, (4) esophagus, and (5) undefined normal tissue. These priorities are reflected in the wish-list applied for automated planning (1). A list of planning objectives is given in Table S1.

Table S1. Planning objectives for the PTV, OARs and normal tissue. D_p = prescribed dose.

Volume	Dose objective
PTV	$V_{95\%} > 98\%$
Lungs	$V_{5Gy} < 65\%$
	$V_{20Gy} < 35\%$
	$D_{mean} < 20$ Gy
Heart	$V_{30Gy} < 40\%$
Esophagus	$D_{mean} < 34$ Gy
Spinal canal	$D_{max} < 50$ Gy
Brachial plexus	$D_{max} < 66$ Gy
Patient body	$D_{max} < D_p \cdot 1.07$

S2 NTCP calculations

S2.1 Radiation pneumonitis (RP)

The NTCP for RP grade ≥2 was calculated using a QUANTEC model refined by Appelt et al. (2,3):

$$NTCP = \frac{1}{1+e^{-S}} \text{ with } S = -4.12 + 0.138 \cdot MLD - 0.3711 \cdot (Former\ smoker) - 0.478 \cdot (Current\ smoker) + 0.8198 \cdot (Co-morbidity) + 0.6259 \cdot (Tumor\ location) + 0.5068 \cdot (Old\ age) + 0.47 \cdot (Sequential\ chemo),$$

where MLD is the mean lung dose in Gy and the other parameters are assigned value 1 or 0 according to Table S2.

Table S2. Variables in the NTCP model for radiation pneumonitis.

Variable	Value = 1	Value = 0
Former smoker	Yes	Never smoked/active smoker
Current smoker	Yes	Never smoked/stopped smoking
Co-morbidity [1]	Yes	No
Tumor location	Middle/lower lobe	Upper lobe
Old age	≥63 years	<63 years
Sequential chemo	Yes	No

[1] Chronic obstructive pulmonary disease or other pre-existing lung disease.

S2.2 2-year mortality (heart model)

The NTCP for 2-year mortality based on heart dose was calculated using a model developed by Defraene et al. and revised after external validation in several patient cohorts (3,4):

$$NTCP = \frac{1}{1+e^{-S}} \text{ with } S = -1.3409 + 0.0590 \cdot \sqrt{GTV} + 0.2635 \cdot \sqrt{MHD},$$

where GTV is the combined GTV volume of the primary tumor and nodes in cm^3 and MHD is the mean heart dose in Gy. The GTV volume from the DIBH CT was used in the calculations for both FB and DIBH, as delineation on the DIBH CT was regarded more accurate than on the AIP (the average difference in GTV volume between DIBH and FB was 2.7%, with DIBH volumes being slightly larger).

S2.3 Acute esophageal toxicity (AET)

The NTCP for AET grade ≥2 was calculated using a model developed by Wijsman et al. and revised after external validation in several patient cohorts (3,5):

$$NTCP = \frac{1}{1+e^{-S}} \text{ with } S = -3.634 + 1.496 \cdot \ln(MED) - 0.0297 \cdot OTT,$$

where MED is the mean esophagus dose in Gy and OTT is the overall radiotherapy treatment time in days.

S2.4 2-year mortality (EDIC model)

The effective radiation dose to immune cells (EDIC) in circulating blood, estimated as the equivalent uniform dose to the entire blood during the radiotherapy course, and the corresponding 2-year overall survival (OS) were calculated based on the models of Jin et al. (6), with some adjustments to accommodate the DIBH scenario:

$$EDIC = B_1\% \cdot MLD + B_2\% \cdot MHD + \left[B_3\% + B_4\% \cdot k_1 \cdot \left(\frac{n}{45}\right)^{\frac{1}{2}}\right] \cdot MBD$$

where $B_1\% = 0.12$, $B_2\% = 0.08$, $B_3\% = 0.45$ and $B_4\% = 0.35$ represent the percentages of the total blood volume contained in the lungs, heart, great vessels, and small vessels in other organs, respectively, MLD, MHD and MBD are the mean doses to the lungs, heart and body, respectively, $k_1 = 0.85$ is a dose effectiveness factor due to the small percentage of cardiac output for the small vessels, and n is the number of fractions.

The mean body dose was calculated as $MBD = ID/(61.8 \cdot 10^3)$, where $61.8 \cdot 10^3$ cm^3 is the estimated average body volume and ID is the integral dose to the body. The ID was not straightforwardly calculated in the DIBH vs. FB scenario. The increased lung volume leads to more air and more body volume around the treatment area and therefore more low dose in the patient body (Figure S1). This dose is, however, given to air; there is not more tissue or blood in the lungs. To correct for this, based on the fact that the amount of functional lung tissue is the same in FB and DIBH, a constant value approximating the lung volume was used for both techniques. To this purpose, the ID was split in two parts: the integral dose in the body volume included in the CT scan

minus the lungs, $M(B-L)D \cdot V(B-L)$, plus the integral dose in the lungs, $MLD \cdot V(L)$, where the average FB value of 3800 cm^3 was used as the lung volume in both FB and DIBH for all patients.

The integral dose in the whole patient was approximated as the integral dose in the body included in the CT scan. Because the CT scans always included the whole lungs and due to limitations in the 4DCT scan length depending on the patient's breathing frequency, the DIBH scan was usually longer than the FB scan for each patient. Therefore, more of the very low doses to the patient body were included in the dose statistics with DIBH. This difference was mainly seen for doses <0.5 Gy. Doses below this were therefore not included when calculating the ID. This had only a small impact on the calculated EDIC (-0.4% for FB and -0.6% for DIBH).

The 2-year OS based on the EDIC was calculated as

$$OS = 0.74 \cdot \left[1 - \frac{0.39}{1+\left(\frac{4.5}{EDIC}\right)^{6}}\right] \cdot \left[1 - \frac{1}{1+\left(\frac{9.9}{EDIC}\right)^{12}}\right],$$

with the resulting NTCP for 2-year mortality: $NTCP = 1 - OS$.

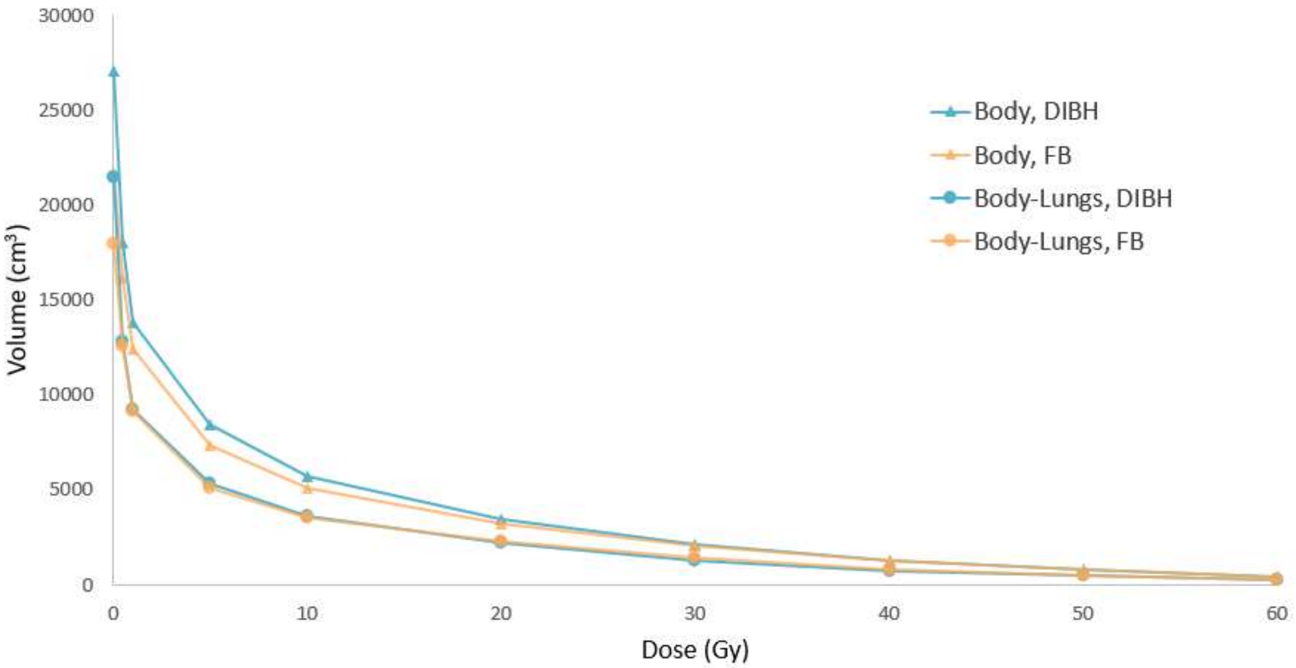

Figure S1. Average DVHs for the patient body and body without lungs in FB and DIBH plans. The figure illustrates that there is an increase in the absolute body volume receiving low doses with DIBH, but that this is solely due to the increased lung volume (more air in the lungs). The volume receiving very low doses (<0.5 Gy) is larger with DIBH due to increased scan length.

S3 Robustness of the dose distribution

Table S3. Dose-volume metrics for the planning FB plans, and the recalculated plans on the FB CTs in W1 and W3. Median value and 10th-90th percentile (pctl) is given for each time point, along with *p*-values for comparison with planning. Significant differences are marked with *. The 35 patients who completed all three CT scans are included.

	**FB planning**		**FB W1**		***p*-value, planning vs. W1**	**FB W3**		***p*-value, planning vs. W3**
	**Median**	**10th–90th pctl**	**Median**	**10th–90th pctl**		**Median**	**10th–90th pctl**	
CTV $V_{95\%}$ (%)	100	–	100	98.0–100	<0.001 *	100	98.9–100	<0.001 *
Patient D_{max} (%)	104.9	103.9–106.0	105.6	103.8–109.1	<0.001 *	106.8	104.3–109.7	<0.001 *
Lungs D_{mean} (Gy)	15.0	9.2–19.2	15.5	10.0–19.6	0.4	15.1	9.8–19.6	0.03 *
Lungs V_{5Gy} (%)	58.7	41.0–79.8	59.8	41.6–78.6	0.7	59.6	41.6–79.2	0.09
Lungs V_{20Gy} (%)	24.7	15.0–34.8	24.4	16.1–34.8	0.5	25.0	15.7–35.7	0.09
Heart D_{mean} (Gy)	8.5	2.6–21.2	8.9	2.3–21.9	0.9	10.0	2.0–20.5	1.0
Heart V_{5Gy} (%)	42.8	9.5–88.0	39.0	8.8–91.4	0.9	42.7	7.7–87.7	1.0
Heart V_{30Gy} (%)	8.6	1.3–23.5	8.3	1.0–25.0	0.9	8.0	0.7–22.1	1.0
Esophagus D_{mean} (Gy)	19.8	10.7–31.0	20.3	10.6–30.3	0.5	21.2	11.7–33.0	0.005 *
Esophagus V_{20Gy} (%)	37.6	22.9–55.9	38.9	17.2–55.3	0.6	41.7	24.9–58.6	0.002 *
Esophagus $V_{60\ Gy}$ (%)	5.2	0.0–29.1	4.7	0.1–29.6	0.03 *	7.2	0.0–34.6	0.01 *
Spinal canal D_{max} (Gy)	46.5	33.8–51.1	46.2	34.4–51.8	0.9	46.8	34.3–52.6	0.07
EDIC (Gy)	4.6	2.9–6.8	4.7	2.8–6.7	0.6	4.7	3.0–7.0	0.1
NTCP RP (%) [1]	20.3	8.2–38.1	21.9	6.8–39.4	0.4	21.2	7.3–37.5	0.03 *
NTCP AET (%) [2]	39.3	21.3–55.4	39.3	20.4–54.6	0.5	43.3	21.1–57.1	0.01 *
NTCP Mortality_Heart (%) [3]	51.4	37.1–66.2	50.1	37.0–66.0	0.9	50.8	37.0–68.0	0.8
NTCP Mortality_EDIC (%) [4]	41.7	27.8–53.3	42.0	27.6–52.9	0.8	42.1	28.1–53.7	0.1

[1] Radiation pneumonitis grade ≥2, [2] acute esophageal toxicity grade ≥2, [3] 2-year mortality (heart model), [4] 2-year mortality (EDIC model).

Table S4. Dose-volume metrics for the planning DIBH plans, and the recalculated plans on the DIBH CTs in W1 and W3. Median value and 10th-90th percentile (pctl) is given for each time point, along with *p*-values for comparison with planning. Significant differences are marked with *. The 35 patients who completed all three CT scans are included.

	**DIBH planning**		**DIBH W1**		***p*-value, planning vs. W1**	**DIBH W3**		***p*-value, planning vs. W3**
	**Median**	**10th–90th pctl**	**Median**	**10th–90th pctl**		**Median**	**10th–90th pctl**	
CTV $V_{95\%}$ (%)	100	99.9–100	100	98.0–100	0.007 *	100	99.1–100	0.002 *
Patient D_{max} (%)	104.7	104.0–106.0	106.6	103.8–109.2	<0.001 *	107.0	105.5–109.9	<0.001 *
Lungs D_{mean} (Gy)	13.5	7.5–17.2	13.7	7.8–17.2	0.5	13.5	7.8–17.3	0.02 *
Lungs V_{5Gy} (%)	55.5	36.0–74.2	55.1	36.0–74.1	1.0	55.6	35.9–74.4	0.07
Lungs V_{20Gy} (%)	22.5	12.2–31.4	22.6	12.5–31.1	0.8	22.9	12.4–31.8	0.07
Heart D_{mean} (Gy)	7.9	1.5–19.6	8.2	1.6–20.3	0.6	9.2	1.6–19.7	0.6
Heart V_{5Gy} (%)	31.3	4.9–94.8	32.4	5.0–96.7	0.2	34.9	5.3–94.6	0.3
Heart V_{30Gy} (%)	7.6	0.0–16.7	6.9	0.2–17.6	0.7	7.0	0.2–17.4	0.9
Esophagus D_{mean} (Gy)	19.4	13.0–30.6	19.7	12.3–28.0	0.06	21.9	13.0–32.5	0.003 *
Esophagus V_{20Gy} (%)	37.5	25.9–56.4	37.0	24.2–62.7	0.1	40.3	27.2–61.2	0.01 *
Esophagus $V_{60\ Gy}$ (%)	5.7	0.0–24.4	6.4	0.0–22.1	0.8	9.2	0.0–27.3	0.001 *
Spinal canal D_{max} (Gy)	42.9	29.1–50.9	43.3	29.9–52.7	0.4	44.4	30.6–53.0	0.02 *
EDIC (Gy)	4.4	2.7–6.2	4.1	2.7–6.4	0.4	4.4	2.6–6.3	0.1
NTCP RP (%) [1]	18.3	6.9–32.7	18.2	6.8–34.1	0.4	18.5	6.8–34.4	0.009 *
NTCP AET (%) [2]	39.2	23.9–56.1	38.6	22.1–53.9	0.04 *	42.0	26.5–58.4	0.005 *
NTCP Mortality_Heart (%) [3]	50.9	36.5–64.5	49.1	37.2–64.4	0.7	50.7	37.2–65.1	0.6
NTCP Mortality_EDIC (%) [4]	39.5	27.2–51.2	36.9	27.2–51.9	0.7	39.6	27.1–51.8	0.2

[1] Radiation pneumonitis grade ≥2, [2] acute esophageal toxicity grade ≥2, [3] 2-year mortality (heart model), [4] 2-year mortality (EDIC model).

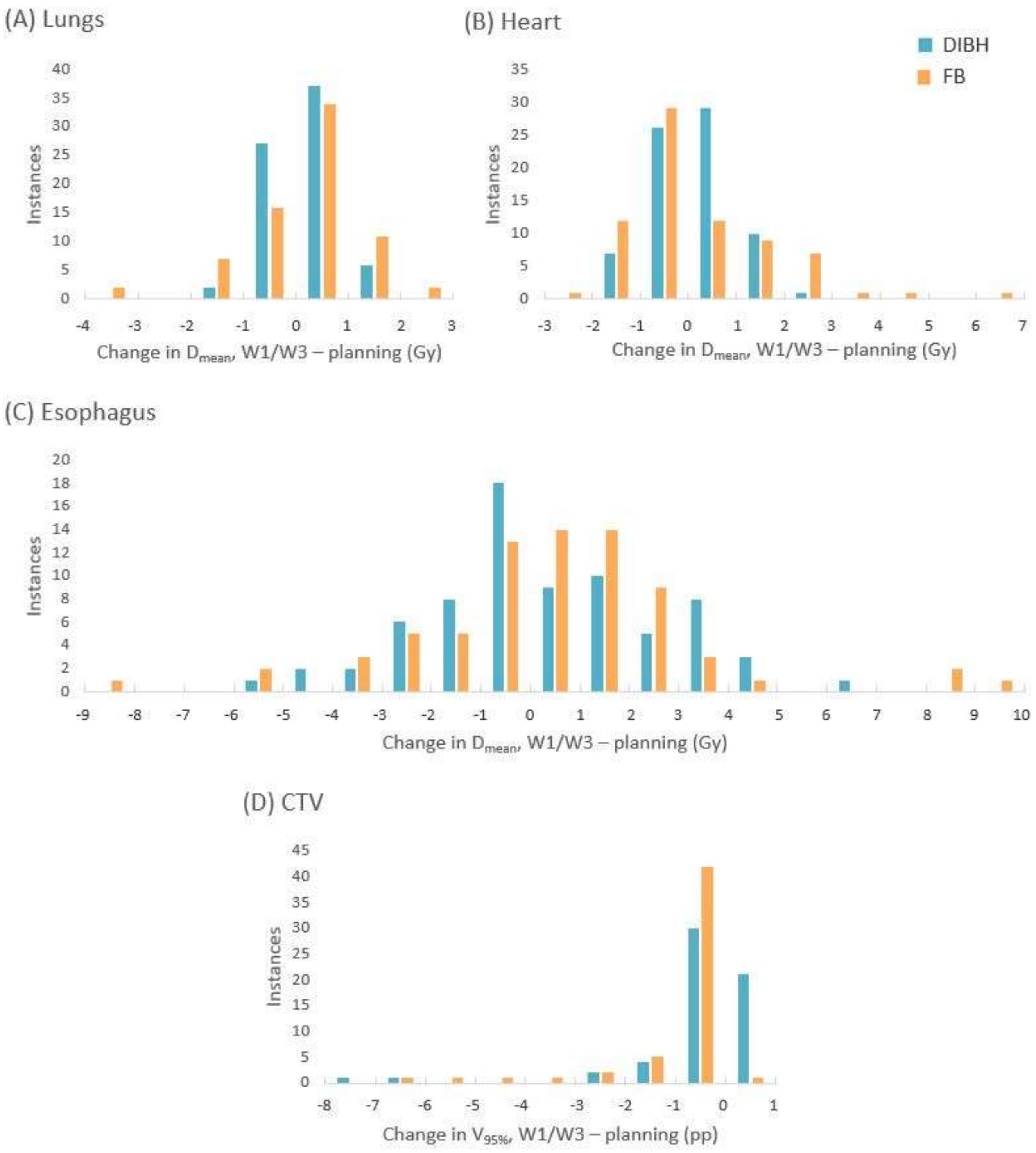

Figure S2. Intra-patient changes in the mean doses to (A) lungs, (B) heart and (C) esophagus, and (D) CTV $V_{95\%}$, from planning to week 1 and planning to week 3 evaluated together. pp = percentage points.

S4 Patient characteristics and benefit of DIBH

Table S5. Average ΔNTCP between DIBH and FB for radiation pneumonitis grade ≥2 (RP), acute esophageal toxicity grade ≥2 (AET) and 2-year mortality with the heart dose and EDIC models for patients with primary tumor in the upper or lower lobe and left or right lung. Negative ΔNTCP values are in favor of DIBH and positive values are in favor of FB. *p*-values are given for comparison between FB and DIBH within each group, and significant differences are marked with *.

	**Upper lobe (n=20)**			**Lower lobe (n=17)**			**Left lung (n=16)**			**Right lung (n=21)**		
	**Avg**	**STD**	***p***	**Avg**	**STD**	***p***	**Avg**	**STD**	***p***	**Avg**	**STD**	***p***
ΔNTCP, RP (pp)	-3.7	3.4	<0.001 *	-4.1	3.2	0.001 *	-3.2	2.9	0.001 *	-4.4	3.5	<0.001 *
ΔNTCP, AET (pp)	0.5	5.0	0.7	-0.3	6.0	0.5	2.2	6.4	0.2	-1.5	4.1	0.09
ΔNTCP, Mortality_Heart (pp)	-1.3	1.5	0.001 *	-0.4	2.0	0.3	-1.5	1.8	0.007 *	-0.5	1.7	0.1
ΔNTCP, Mortality_EDIC (pp)	-2.6	2.2	<0.001 *	-2.2	1.9	0.001 *	-2.1	1.9	0.001 *	-2.6	2.2	<0.001 *

Table S6. Results from the linear regressions between the ΔNTCPs for radiation pneumonitis grade ≥2 (RP), acute esophageal toxicity grade ≥2 (AET) and 2-year mortality with the heart dose and EDIC models vs. the cranio-caudal motion of the primary tumor GTV in FB, and vs. the expansion of the lungs with DIBH. The reported parameters are the intercept (a), slope (b), coefficient of determination (r^2) and *p*-value of the linear regression. There were no statistically significant correlations.

	**Tumor motion**				**Lung expansion**			
	**a**	**b**	**r^2**	***p***	**a**	**b**	**r^2**	***p***
ΔNTCP, RP (pp)	-3.0	-0.2	0.07	0.1	-1.5	-0.04	0.06	0.2
ΔNTCP, AET (pp)	0.9	-0.2	0.02	0.4	-1.9	0.04	0.02	0.5
ΔNTCP, Mortality_Heart (pp)	-1.2	0.06	0.03	0.3	-1.0	0.003	0.001	0.9
ΔNTCP, Mortality_EDIC (pp)	-2.1	-0.1	0.02	0.4	-1.2	-0.02	0.04	0.3

References

1. Fjellanger K, Hysing LB, Heijmen BJM, Pettersen HES, Sandvik IM, Sulen TH, et al. Enhancing Radiotherapy for Locally Advanced Non-Small Cell Lung Cancer Patients with iCE, a Novel System for Automated Multi-Criterial Treatment Planning Including Beam Angle Optimization. Cancers. 2021 Nov 13;13(22):5683.

2. Appelt AL, Vogelius IR, Farr KP, Khalil AA, Bentzen SM. Towards individualized dose constraints: Adjusting the QUANTEC radiation pneumonitis model for clinical risk factors. Acta Oncologica. 2014 May;53(5):605–12.

3. Nederlandse Vereniging voor Radiotherapie en Oncologie. Landelijk Indicatie Protocol Protonen Therapie - Longcarcinoom [Internet]. 2019 Jun. Available from: nvro.nl/images/documenten/rapporten/LIPP_longen_final_01122019.pdf

4. Defraene G, Dankers FJWM, Price G, Schuit E, van Elmpt W, Arredouani S, et al. Multifactorial risk factors for mortality after chemotherapy and radiotherapy for non-small cell lung cancer. Radiotherapy and Oncology. 2020 Nov;152:117–25.

5. Wijsman R, Dankers F, Troost EGC, Hoffmann AL, van der Heijden EHFM, de Geus-Oei LF, et al. Multivariable normal-tissue complication modeling of acute esophageal toxicity in advanced stage non-small cell lung cancer patients treated with intensity-modulated (chemo-)radiotherapy. Radiotherapy and Oncology. 2015 Oct;117(1):49–54.

6. Jin JY, Hu C, Xiao Y, Zhang H, Paulus R, Ellsworth SG, et al. Higher Radiation Dose to the Immune Cells Correlates with Worse Tumor Control and Overall Survival in Patients with Stage III NSCLC: A Secondary Analysis of RTOG0617. Cancers. 2021 Dec 8;13(24):6193.
